# Hydrophobins in the Life Cycle of the Ectomycorrhizal Basidiomycete *Tricholoma vaccinum*

**DOI:** 10.1371/journal.pone.0167773

**Published:** 2016-12-09

**Authors:** Dominik Sammer, Katrin Krause, Matthias Gube, Katharina Wagner, Erika Kothe

**Affiliations:** Institute of Microbiology, Friedrich Schiller University Jena, Jena, Germany; University of Wisconsin Madison, UNITED STATES

## Abstract

Hydrophobins—secreted small cysteine-rich, amphipathic proteins—foster interactions of fungal hyphae with hydrophobic surfaces, and are involved in the formation of aerial hyphae. Phylogenetic analyses of *Tricholoma vaccinum* hydrophobins showed a grouping with hydrophobins of other ectomycorrhizal fungi, which might be a result of co-evolution. Further analyses indicate angiosperms as likely host trees for the last common ancestor of the genus *Tricholoma*. The nine hydrophobin genes in the *T*. *vaccinum* genome were investigated to infer their individual roles in different stages of the life cycle, host interaction, asexual and sexual development, and with respect to different stresses. In aerial mycelium, *hyd8* was up-regulated. *In silico* analysis predicted three packing arrangements, i.e., ring-like, plus-like and sheet-like structure for Hyd8; the first two may assemble to rodlets of hydrophobin covering aerial hyphae, whereas the third is expected to be involved in forming a two-dimensional network of hydrophobins. Metal stress induced hydrophobin gene *hyd5*. In early steps of mycorrhization, induction of *hyd4* and *hyd5* by plant root exudates and root volatiles could be shown, followed by *hyd5* up-regulation during formation of mantle, Hartig’ net, and rhizomorphs with concomitant repression of *hyd8* and *hyd9*. During fruiting body formation, mainly *hyd3*, but also *hyd8* were induced. Host preference between the compatible host *Picea abies* and the low compatibility host *Pinus sylvestris* could be linked to a stronger induction of *hyd4* and *hyd5* by the preferred host and a stronger repression of *hyd8*, whereas the repression of *hyd9* was comparable between the two hosts.

## Introduction

The mutualistic ectomycorrhizal symbiosis of basidiomycetes with tree roots plays an important role in forest ecosystems, where trees are connected *via* fungal mycelia leading to better nutrient and water distribution [1]. Based on the interaction, the plant benefits from mineral supply, resistance against pathogens, growth promotion and increased metal tolerance, whereas the fungus is supported with growth promoting factors, vitamins and up to 30% of net photosynthetic carbohydrates (for review [2]). More than 50% of the mRNAs were differentially expressed upon host interaction in *Pisolithus tinctorius* [3]. Knowledge on ectomycorrhiza (ECM) increased significantly, when studies of gene expression, carbohydrate metabolism or nitrogen transport became available for different ectomycorrhizal fungi. Signaling involved in host recognition, e.g. by abietic acid, oligopeptides, phytohormones, volatiles, and hydrophobins, has been discussed (for reviews, see [2, 4, 5] and citations therein).

More than 6,000 fungal species are estimated to form ECM, with the fungus functionally replaces root hairs by hyphae extending into the surrounding soil, coating the short roots with a pseudoparenchymatic mantle, and penetrating into the extracellular space to form the characteristic Hartig’ net, where the exchange of nutrients between both partners takes place (for review [2]).

The genus *Tricholoma* is widely distributed, with 90–100 species world-wide, 65–70 of which are found in Europe [6]. These basidiomycetes typically form late stage ECM, associated with slow growth and high adaptive capacity leading to high host specificity. Within the genus, three clades are separated, easily distinguishable by their cap colours with brown, yellow and white/grey capped *Tricholoma* species [7]. Their mode of living includes saprotrophic growth on plant litter and ectomycorrhiza developed on diverse gymnosperm and angiosperm host trees including *Pinus*, *Picea*, *Fagus* and *Quercus*. Only after the symbiotic association is established, the fungus is capable to form fruiting bodies. However, the signals that trigger the formation of fruiting bodies remain elusive. The species *T*. *vaccinum* undergoes mutualistic symbiotic interactions with conifers, predominantly spruce, and rarely pine [6, 8]. In axenic co-cultures host specificity is seen with compatible interactions established within weeks, while low compatibility interactions need several months for development [8]. In nature, 98% of the harvested fruiting bodies are found under the native host spruce (*Picea abies*), while 2% occur near the low compatibility host *Pinus sylvestris* [6]. Using co-cultures, differential gene expression was analyzed with respect to mycorrhization and host specificity [9].

The mediators of interaction between host and fungus in mycorrhiza can be either volatiles or secreted proteins and small organic molecules. Signalling molecules of the plant can be expected in the released exudates that include carbohydrates, low molecular weight aliphatic and aromatic acids, fatty acids, enzymes, phytohormones like indole-3-acetic acid, and amino acids like tryptophan, or in the volatiles that may contain ethanol, acetaldehyde and monoterpenes like 1,8-cineole [4]. Different chemical composition of tree root exudates, *e*.*g*. organic acids, are reported for conifers *P*. *abies* and *P*. *sylvestris* also in relation to environmental conditions [10, 11]. Additionally, ethanol is known to be accumulated in roots especially during hypoxia [12]. Volatiles of both, the tree and the fungus, include terpenes like pinenes, camphene and limonene, 1-octen-3-ol and 3-octenone [13], and fungus derived sesquiterpenes affect root architecture [13, 14]. Plant-derived phytohormones were shown to increase growth and hyphal ramification in *T*. *vaccinum*, and the fungus is able to produce indole-3-acetic acid [8, 15].

Hydrophobins are small, secreted proteins, about 100 amino acids long, with low sequence homologies. They cluster into class I (basidiomycetes) and class II (mainly ascomycetes) with respect to their solubility; and all contain a consensus of eight cysteine residues’ spacing, with disulfide bridges being formed [16]. The first hydrophobin characterized was Sc3 from *Schizophyllum commune*, a white rot basidiomycete. This protein plays an essential role in formation of aerial mycelium. Structure predictions as well as pictures showing the formation of rodlet like structures formed on aerial hyphae allowed for an identification of structure-function association in this well-studied system [17–21]. After secretion of soluble monomers into the medium, it aggregates and covers the hydrophilic hyphae upon conformational change at hydrophilic/hydrophobic interfaces. The lowered surface tension allows hyphae to breach the surface and grow into the air. A Δ*sc3* mutant lacks aerial mycelium, and is easily wettable [22]. A further characteristic of hydrophobins covering aerial hyphae is their ability to form rodlet structures, with class I aggregates being smaller in size (7–15 nm), insoluble in water and harder to dissolve as compared to class II hydrophobins [23, 24].

The protein Sc3 is posttranslationally modified by O-glycosylation at Thr19 and Ser42 with addition of 16 to 22 mannosyl residues [25]. Similar glycosylation was predicted for Hyd1 of *T*. *terreum*, since the anti-Sc3 antibody recognized primarily the glycosylated molecular pattern [26]. Using immuno-fluorescence imaging, a host-specific enrichment of this hydrophobin protein in the Hartig’ net during a compatible interaction was shown with the native host *P*. *sylvestris*, while the low compatibility interaction with spruce showed expression only with hyphae in the mantle of *Tricholoma* mycorrhizal roots [26].

With *S*. *commune*, other hydrophobin genes were shown to be present only in fruiting bodies (Sc1 and Sc4; [27]). Differential roles for different hydrophobins thus is already established with a system that is genetically tractable. With respect to *Tricholoma*, different roles of single hydrophobins might explain the host or substrate specificity [26], and further hypothetic functions might be inferred from differential expression, as was the case with the *S*. *commune* proteins. Biotic and abiotic factors affect the regulation of hydrophobin genes [28, 29]. In *S*. *commune* as well as in lichens, a specifically expressed hydrophobin lines air channels of fruiting bodies or thalli [30]. Changes in the environment lead to hydrophobin repression, *e*.*g*. exposure to cadmium that was suggested to cause a re-direction of metabolic fluxes in *Paxillus involutus* with cysteine being used to form, e.g., glutathione that is important for complexation and detoxification of heavy metals [31]. Functions of hydrophobins include regulating cell wall integrity, conidiospore hydrophobicity (*dewA* of *Aspergillus nidulans*), adhesion in pathogenic interactions (*mpg1* of *Magnaporthe grisea*), as well as ectomycorrhizal interactions [32].

However, the biological function of hydrophobins is not strictly defined. With this study, a structure prediction using *in silico* analyses was performed to elucidate the relation between structure and function for aerial hyphae formation of one of the encoded hydrophobins that is found up-regulated in aerial hyphae. Numbers of genes coding for hydrophobins in different basidiomycetes differ, and a probable functional specialization of the different members of the family has been proposed [33]. Here, we aimed at investigating differential expression of hydrophobins using of all nine hydrophobin genes of *T*. *vaccinum* [34]. The expression patterns allowed us to infer potential roles for each of the hydrophobins in this fungus which mainly grows as a dikaryon. Previous efforts to produce monokaryons by protoplasting were not successful and the absence of auxotrophic mutants prevents knock-out analyses. The present work refines our knowledge on hydrophobin regulation, especially with regard to ECM that impacts ecology of forest growth and woodland ecosystem function and stability, as well as the processes involved in establishing the mycorrhizal association.

## Materials and Methods

### Organisms and culture conditions

The *Tricholoma* strains used in this study were obtained from the Jena Microbial Resource Center (JMRC, see Supporting Table 1 in S1 File) or by collecting fully grown, fresh fruiting bodies around Jena, Germany (+50° 55’ 11.29, +11° 31’ 30). The genome sequence of *T*. *vaccinum* GK6514 [34] was used to obtain hydrophobin sequences from all strains (NCBI GenBank accession numbers: Supporting Table 2 in S1 File).

For DNA or RNA extraction from fruiting bodies, the samples were ground in liquid nitrogen within 1 h of collection. To exclude contamination, ITS PCR was performed [35].

*T*. *vaccinum* was cultivated on half-concentrated MMNb [36]. To induce substrate mycelium on soil containing solid media, 10 g/l of commercially available potting soil (COMPO GmbH, Germany) was diluted in distilled water. For metal treatments, 20 d liquid cultures in half-concentrated MMNb were treated for 24 h with sublethal concentration of 0.5 mM NiCl_2_, CaCl_2_, CuCl_2_ and 0.05 mM CdCl_2_. The sublethal concentrations had been established by a dilution series that defined inhibitory effects to fungal growth as a dikaryon (data not shown). The time of 24 h was necessary since the (non-synchronous) fungal cells are slow-growing at > 12 h doubling times. Further, we could not record substantial differences in gene expression in shorter time spans.

Seeds (Landesforst Mecklenburg-Vorpommern, Germany) were germinated and grown as previously described [9]. Root exudates of ten one-month-old plants of *P*. *abies* or *P*. *sylvestris* were collected under sterile conditions by transferring plantlets into 1 ml sterile pipette tips of a sterile pipette tip box (standard rack, Greiner Bio-One GmbH, Frickenhausen, Germany) containing 100 ml 20% MMNa solution (Supporting Figure 1 in S1 File [36, 37]). The incubation was performed in a moisture chamber. After one week of incubation, the liquid was filtered (Millipore 0.22 μm, Millipore) and 70 ml half-concentrated MMNb of a 20 d liquid culture was treated for 24 h with 15 ml of root exudate before extracting RNA. A treatment for 24 hours was necessary to induce gene regulation of the slow-growing (non-synchronous) fungal cells and to have a substantial portion of induced hyphae.

For the investigation of potential active substances known from root exudates, indole-3-acetic acid (IAA) and ethanol were tested. A pre-grown liquid culture (20 d, half-concentrated MMNb) was supplemented with 0.01% ethanol or IAA (0.1, 0.01, 0.001 mM) dissolved in 0.01% ethanol for 24 hours. For the early transcriptional answer of the mycobiont to the exposure to root volatiles, *T*. *vaccinum* was cultivated with host on one side containing half-concentrated MMNb of a two-compartment split Petri dish on a cellophane membrane (Wilhelm Isermann KG, Walsrode, Germany) either for 4 or for 20 days. The plant was placed on MMNa medium with root inside, stipe and leaf outside of the dish (Supporting Figure 1 in S1 File).

An axenic Petri dish system was used to synthesize ectomycorrhiza (Supporting Figure 1 in S1 File) as previously described by Krause and Kothe [9], but here the complete seedling was enclosed in the Petri dish. The system was incubated for 6–8 weeks, until the typical morphology of ectomycorrhizal root tips was observed. The mycorrhized area of the root was harvested, aerial mycelium removed and total RNA extracted.

### Quantitative real-time PCR

Because of low biomass production of the fungus, at least three independent biological replicates were combined, and two technical replicates for each RNA isolation were performed [38] with two reverse transcriptions using 1 μg or 0.1 μg of total RNA (QuantiTect Reverse Transcription Kit, Qiagen). All cDNAs were measured 3 times for each gene (Maxima SYBR Green qPCR Master Mix, Thermo Fisher, Waltham, MA, USA) using a SmartCycler II thermocycler (Cepheid, Sunnyvale, CA, USA). For amplification of the target hydrophobin genes *hyd1* through *hyd9*, as well as the reference genes *act1* (actin) and *tef1* (translation elongation factor EF1α), oligomers were synthesized by Eurofins (Gibco, Karlsruhe, Germany; Supporting Table 3 in S1 File). Neither the actin gene nor the elongation factor responded with differential regulation to the different treatments (Supporting Figure 2 in S1 File [39, 40, 41]). The primers span an intron to identify potential genomic DNA contamination visible in different PCR product lengths, e.g. for *tef1* 150 bp with DNA in comparison to 100 bp with cDNA. Primer efficiencies were calculated based on the slope of a standard curve generated from series of different cDNA concentrations. The data were normalized and quantified relative to the expression of the reference genes and corrected for the calculated efficiency [42, 43]. Unspecific binding of primers to plant RNA and genomic DNA was not observed. For expression analysis of *T*. *vaccinum* fruiting bodies different primers (hyd1-8fb, Supporting Table 3 in S1 File) were used according to the minor changes in the nucleotide sequence. For the data analysis the efficiency of the primers was set to 100%.

### ITS and hydrophobin PCR

Isolated genomic DNA [44] was used for ITS1/ITS4 PCR [45] with initial denaturation for 120 s at 95°C; 45 cycles of denaturation for 30 s at 95°C, annealing for 30 s at 56°C, and extension for 48 s at 72°C and hydrophobin amplification with a gradient PCR (initial denaturation for 120 s at 95°C, 45 cycles of denaturation for 30 s at 95°C, annealing for 30 s at 52–58°C, and extension for 30 s at 72°C; with primers hyd1-9ampl, Supporting Table 3 in S1 File). The amplicons were ligated into a cloning vector (pDrive, Qiagen) and sequenced with T7 and SP6 primers (GATC Biotech AG).

ITS sequences were compared against NCBI and UNITE database entries for phylogenetic analyses. Alignments were created with MAFFT (version 7.221 [46] by online server, using Q-INS-i strategy for ITS, default settings for hydrophobin genes and Blossum 80 protein substitution matrix). The program MrBayes (version 3.2) was used for phylogenetic analysis [47, 48]. Prediction of potential glycosylation sites was performed (http://www.imtech.res.in/raghava/glycoep).

### Phylogenetic analyses

For all datasets, two runs with each 1,000,000 generations in four chains were performed, with sampling every 100 generations, and with a burn-in of 20 percent (GTR+G was assumed for ITS sequences, Dayhoff model for hydrophobin protein sequences). Full length hydrophobin gene and mature protein sequences were combined to reconstruct a phylogenetic tree. In order not to confuse the lineages within basidiomycetes by rooting the tree in one of them, the outgroup class II hydrophobin of *Paracoccoides brasiliensis* was chosen.

BayesTraits version 2.0 [49] was used to obtain ancestral states by Maximum Likelihood (default) and Bayesian method (default, burnin 50000). The calculation of abundance of *Tricholoma* under a specific host tree was adopted from Kriegelsteiner [6]. To allow for calculation of ratios, the numbers were classified from percent into classes (A = 0%; B = 1–24%; C = 25–49%; D = 50–74%; E = 75–99%; F = 100%, Supporting Table 4 in S1 File). Bayesian results were analyzed with TRACER version 1.5 [50] and visualized with FigTree version 1.4.2 [51]. The rates of nonsynonymous (*d*_*n*_) and synonymous (*d*_*s*_) nucleotide substitutions per site for each of the orthologous groups were estimated separately using the CRANN software [52] with the hydrophobin 1 gene of *Moniliophthora roreri* MCA 2997 (XM_007854886) as outgroup. Hydrophobicity was predicted with ProtScale [53] with the Kyte & Doolittle scale [54]. Secretion signals were checked with SignalP (version 3.0 [55]) and glycosylation sites with GlycoEP [56]). I-TASSER was used to predict a 3D structure for *T*. *vaccinum* hydrophobins [57] using DewA [58], Hfb2 [59], and EAS [60] as templates with HADDOCK driven prediction to model protein-protein interactions (F86 = active; S85-G89 = passive or F54 = active; V52-D55 = passive; solvent = water [23, 61]). Protein calculator (Innovagen AB, Sweden) was used for the prediction of pH net charge. The similarities of the secondary structure of the hydrophobins were calculated with MATRAS [62]. Amino acid similarities of hydrophobins from *T*. *vaccinum* fruiting body and *T*. *vaccinum* GK6514 were additionally calculated with AlignX (a component of Vector NTI, Invitrogen, 2008) to allow for protein similarity comparisons. To find motifs in mycorrhiza specific genes (*ald1* (HM363121), *mte1* (KP137511), phospholipase [9]) their promoters (-1000 bp to +50 bp) were analyzed with MEME suite [63] and TOMTOM [64] to compare against a database of known DNA motifs.

### Statistical treatments

Fold change values were calculated via ΔΔCt method [38]. To compare groups, a two-tailed unpaired Student’s *T-test* was used for qRT-PCR data on the fold change values. Significance levels were set to * *P-value* < 0.05, ** *P-value* < 0.005, *** *P-value* < 0.005. Groups of data are given as average values ± standard deviation.

## Results

### Class I hydrophobins of *T*. *vaccinum*

Nine hydrophobin genes were identified from a genomic sequence of *T*. *vaccinum* GK6514 based on the lenght of the probable gene, position of introns and the cysteine motif in encoded protein (Table 1). The hydrophobicity pattern of all hydrophobins from *T*. *vaccinum* was consistent with class I hydrophobins (Supporting Figure 3 in S1 File). The *T*. *vaccinum* proteins shared the typical length of hydrophobins with approximately 100 amino acids, except for Hyd7, which features an extended N-terminus (120 amino acids in total). For this protein, as well as for Hyd8, a comparatively high number of putative glycosylation sites were predicted (see Table 1). As expected, all conceptually translated hydrophobins showed secretion signal peptides and the respective consensus for signal sequence proteases (Supporting Figure 4 in S1 File).

**Table 1:** General characteristics of the identified *T*. *vaccinum* hydrophobins.

	Cysteine motif	Potential N- and O-linked glycosylation sites [56]
**Hyd1** (KJ507742)	X_9_-C-X_6_-C-C-X_32_-C-X_13_-C-X_5_-C-C-X_12_-C-X_5_	N: no glycosylation; O: S1
**Hyd2** (KJ507743)	X_9_-C-X_6_-C-C-X_32_-C-X_12_-C-X_5_-C-C-X_12_-C-X_5_	N: no glycosylation; O: T1, T6, S7, (T44, T49)
**Hyd3** (KJ507744)	X_12_-C-X_6_-C-C-X_32_-C-X_17_-C-X_5_-C-C-X_12_-C-X_7_	N: N28; O: T3, (T15, T22, S23, T30, T48)
**Hyd4** (KJ507745)	X_14_-C-X_6_-C-C-X_32_-C-X_18_-C-X_5_-C-C-X_12_-C-X_8_	N: no glycosylation; O: T1, T3, S11, (T21, T26, T50, T51, T60, S61)
**Hyd5** (KJ507746)	X_11_-C-X_6_-C-C-X_32_-C-X_13_-C-X_5_-C-C-X_12_-C-X_5_	N: N78; O: T1, S8, S9, (T51)
**Hyd6** (KJ507747)	X_9_-C-X_6_-C-C-X_32_-C-X_13_-C-X_5_-C-C-X_12_-C-X_5_	N: no glycosylation; O: S1, S5
**Hyd7** (KJ507748)	X_42_-C-X_6_-C-C-X_31_-C-X_12_-C-X_5_-C-C-X_12_-C-X_5_	N: no glycosylation; O: S1, T5, T24, S32, S35, S37, (T55)
**Hyd8** (KJ507749)	X_14_-C-X_6_-C-C-X_32_-C-X_18_-C-X_5_-C-C-X_12_-C-X_8_	N: no glycosylation; O: S6, T10, S11, T13, (T21, S42, T50, T51, T57, T60)
**Hyd9** (KJ507750)	X_13_-C-X_6_-C-C-X_32_-C-X_18_-C-X_5_-C-C-X_12_-C-X_8_	N: N26, N82; O: S41, T49, T50, T59, S60

### Structure and organization of hydrophobin genes of *T*. *vaccinum*

**Fig 1:**
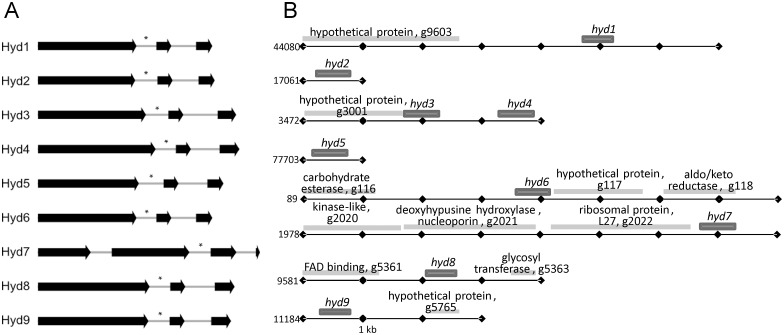
Gene and genomic structure of hydrophobins in *T*. *vaccinum*. (A) The exons of hydrophobin genes are visualized by arrows; the asterisk shows the position of the 5th cysteine residue within a splice site. (B) The genomic structure is presented for the scaffolds of *T*. *vaccinum* genome.

The hydrophobin genes varied in length from 439 bp (*hyd1* and *hyd6*) to 585 bp (*hyd7*) with an average GC-content of 52%. By sequencing of the corresponding cDNAs, two introns at conserved positions for all tested *T*. *vaccinum* isolates with one splice site within the 5^th^ cysteine residue were observed (except for three introns in *hyd7* of *T*. *vaccinum* GK6514 and no intron in *hyd2* of *T*. *vaccinum* MG111121-03, indicative of a late duplication *via* mRNA reverse transcription, Fig 1). The average intron length was typical for basidiomycetes with 61 bp (max 86, min 50 bp). Because of an inverted tandem duplication, the genes *hyd3* and *hyd4* (62% identity) are linked with 1163 bp separating the genes including a shared promoter. Duplication for *hyd1* and *hyd6* with 92% gene and 93% protein identity was more recent. Under no tested condition, *hyd6* mRNA was detectable.

**Fig 2:**
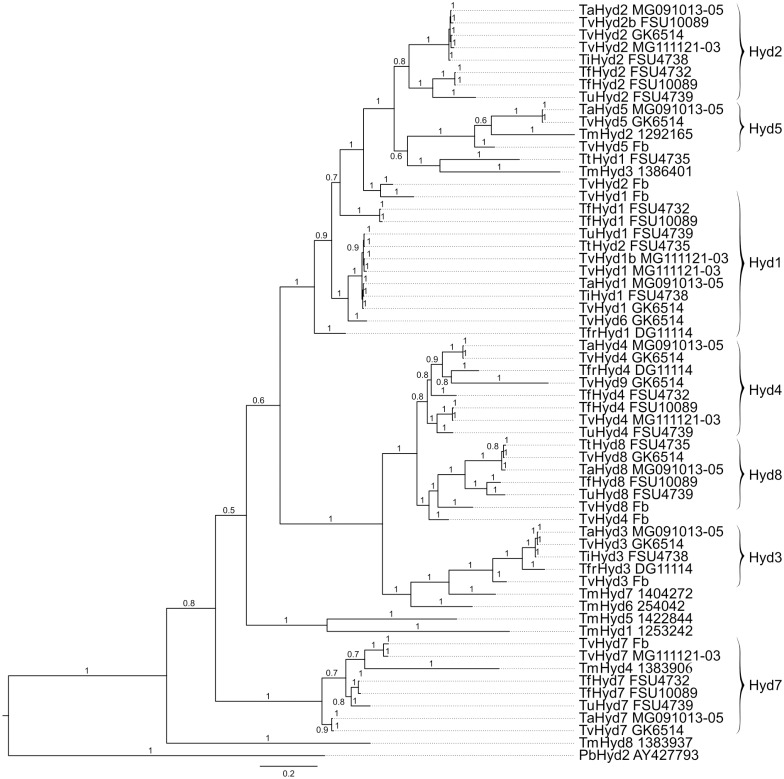
Consensus phylogram of hydrophobins from genus *Tricholoma*. Gene sequences conceptually translated and protein sequences were used from *T*. *albobrunneum* (Ta), *T*. *imbricatum* (Ti), *T*. *fracticum* (Tfr), *T*. *fulvum* (Tf), *T*. *imbricatum* (Ti), *T*. *matsutake* (Tm), *T*. *terreum* (Tt), *T*. *ustaloides* (Tu), *T*. *vaccinum* (Tv), and fruiting body (Fb). Bayesian posterior probability values are shown above corresponding branches; branch lengths are proportional to evolutionary distances. The class II hydrophobin PbHyd2 from the ascomycete *Paracoccidioides brasiliensis* has been included as outgroup.

In total, 43 *Tricholoma* sequences could be identified with 9x *hyd1*, 10x *hyd2*, 4x *hyd3*, 6x *hyd4*, 2x *hyd5*, 6x *hyd7*, 6x *hyd8* (Supporting Table 2 in S1 File). There was no *hyd6* group, as *hyd6* is a duplication of *hyd1* and therefore clusters within this group. In addition, no further *hyd9* could be found in other *Tricholoma* species, indicating a strain dependent duplication (Fig 2). Inverse tandem duplication creating genes *hyd3* and *hyd4* of *T*. *vaccinum* GK6514 is assumed to have resulted from a recent event. The *T*. *vaccinum* hydrophobins except Hyd7 cluster with the closely related species *T*. *terreum* and five (of eight) *T*. *matsutake* and seven (of twelve) hydrophobins of the mycorrhizal fungus *Laccaria bicolor* (Supporting Figure 5 in S1 File). Thus, Hyd7-like hydrophobins form a separate cluster. The gene clustering with potential similar regulatory schemes may be indicative of similar functions. *T*. *matsutake* Hyd7 shows closest sequence similarity, as well as hydrophobicity plot similarity to TvHyd3 of *T*. *vaccinum* and Sc4 of *S*. *commune* with function in fruiting bodies [27]. Within the same branch of hydrophobins, TmHyd6 is orthologous to TvHyd8, TvHyd4 and *S*. *commune* Sc3 (supporting aerial mycelium formation [16]) also visible with pH *versus* net charge profiles (Supporting Figure 6 in S1 File). The host specific *hyd5* group clustered with hydrophobins of the *T*. *vaccinum* fruiting body, *T*. *albobrunneum*, *T*. *terreum* (shown to be involved in host interaction as well [26]) and two sequences of *T*. *matsutake*. Their characteristics in hydrophobicity and net charge *versus* pH are similar. The rates and patterns of nucleotide substitutions were analyzed by calculating the non-synonymous (*d*_n_) and synonymous (*d*_s_) values. Of the 102 pair-wise comparisons, 20 showed a *d*_n_/*d*_s_ ratio > 1, representing a relaxed or positive selection in the Hyd1, Hyd3, Hyd4 and Hyd7 groups.

Since we wanted to relate the hydrophobin lineages to functions in mycorrhization, it was necessary to show relationships to other *Tricholoma* species based on ITS sequences. The phylogeny of *Tricholoma* species shows three main clusters coinciding with different fruiting body colours (Supporting Figure 7 in S1 File). We found the grey-capped *Tricholoma* to be basal, and brown as well as yellow capped species derived. With respect to mycorrhization, the brown and grey *Tricholoma* species are known to mainly form ECM with conifers [6], with only few broadleaf associations being present. The yellow capped species establish mainly strongly compatible ECM interactions with conifers while rarely accepting broadleaved trees such as *Fagus* as low compatibility partners [6]. However, the prediction for the ancestral state of host preference showed associations with angiosperms, *Fagus* and *Betula* and/or *Quercus*. Both methods, the Markov chain Monte Carlo and maximum likelihood predictions, revealed *Fagus* and *Quercus* as being ancestral host trees. In addition, and using only maximum likelihood, a highly compatible association with *Betula* could be seen.

### Regulatory motifs in hydrophobin gene promoters

**Fig 3:**
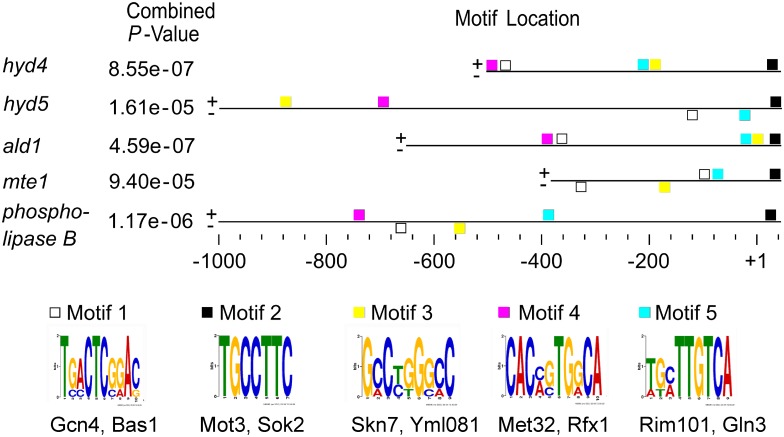
Regulatory motifs of mycorrhiza specific genes. Predictions were obtained from MEME suite and TOMTOM analysis.

To find indications for a regulation based on mycorrhizal interactions and to suggest potential roles for single hydrophobins, we checked the promoter regions of hydrophobin genes in comparison with mycorrhiza specific genes *ald1*, *mte1* and the gene encoding phospholipase B [9] for regulatory motifs (Fig 3). Using the yeast database regulatory elements corresponding to binding regions for,Gcn4 (activator of amino acid biosynthetic genes), Mot3 (repression in hypoxia), Skn7 (response to oxidative stress and involved in osmoregulation), Met32 (transcriptional regulation of methionine biosynthesis and sulfur metabolism), and Rim101 (alkaline response, cell wall assembly, alkaline-stimulated haploid invasive growth and sporulation) were identified.

### Aerial hydrophobins of *T*. *vaccinum*

**Fig 4:**
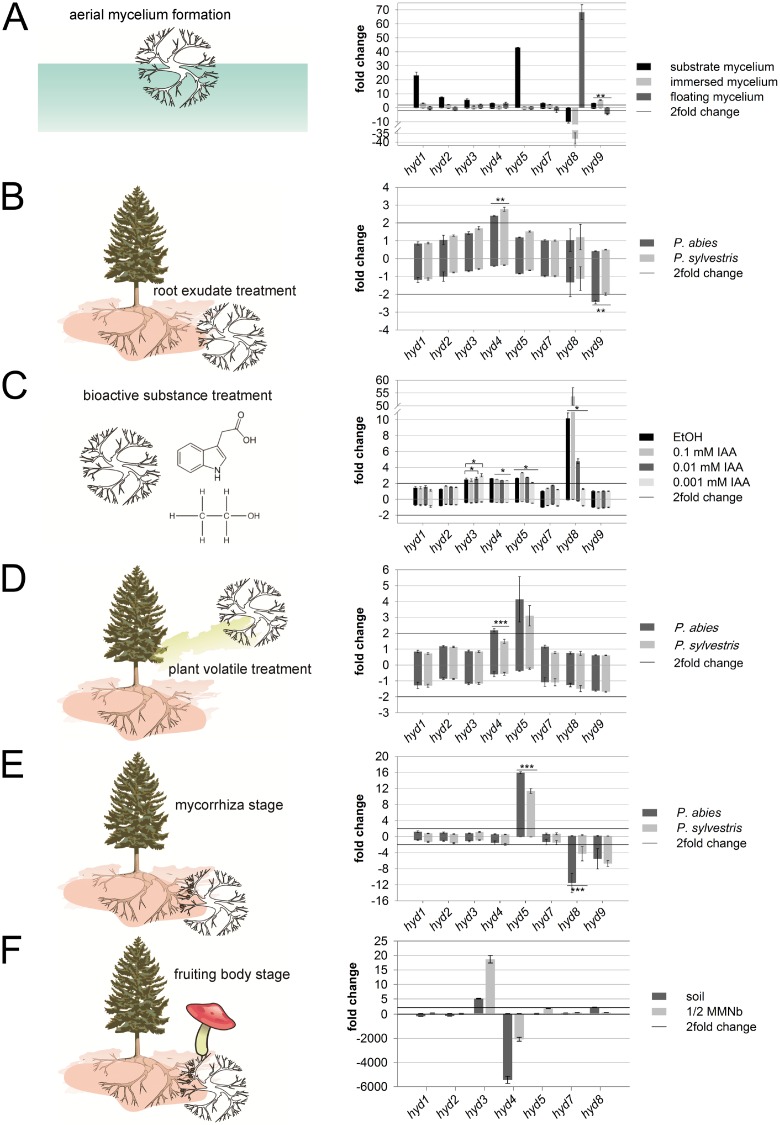
Fold changes of relative expression of hydrophobin transcripts. (A) Mycelium grown on soil (substrate mycelium), immersed and floating mycelium compared to solid medium. (B) Liquid culture treated with root exudates of *P*. *abies* or *P*. *sylvestris*, and (C) with ethanol (EtOH) and indole-3-acetic acid (IAA) both compared with non-treated liquid culture. (D) Response to volatiles from mycelia grown on solid medium, next to roots of *P*. *abies* or *P*. *sylvestris*, and (E) in ectomycorrhiza with *P*. *abies* or *P*. *sylvestris* both compared to a control on solid medium. (F) Fruiting bodies compared to artificial medium and soil. Bars denote standard error, significance level * *P* < 0.05, ** *P* < 0.005, *** *P* < 0.005.

To compare hydrophobin coating of aerial mycelium to substrate mycelium growing on compost soil or submerged in liquid media, expression analyses were performed. Although soil did not show significant induction of aerial mycelium formation, a general induction of hydrophobin genes was seen. This was not the case for *hyd8* that was repressed also in immersed culture, but strongly induced in floating mycelium, while *hyd9* showed the opposite regulation (Fig 4A). In floating mycelium, in addition to *hyd8*, the slight induction of *hyd3* and *hyd4* was observed. This is in accordance with the high number of potential glycosylation sites of Hyd8 as the major aerial mycelium hydrophobin.

**Fig 5:**
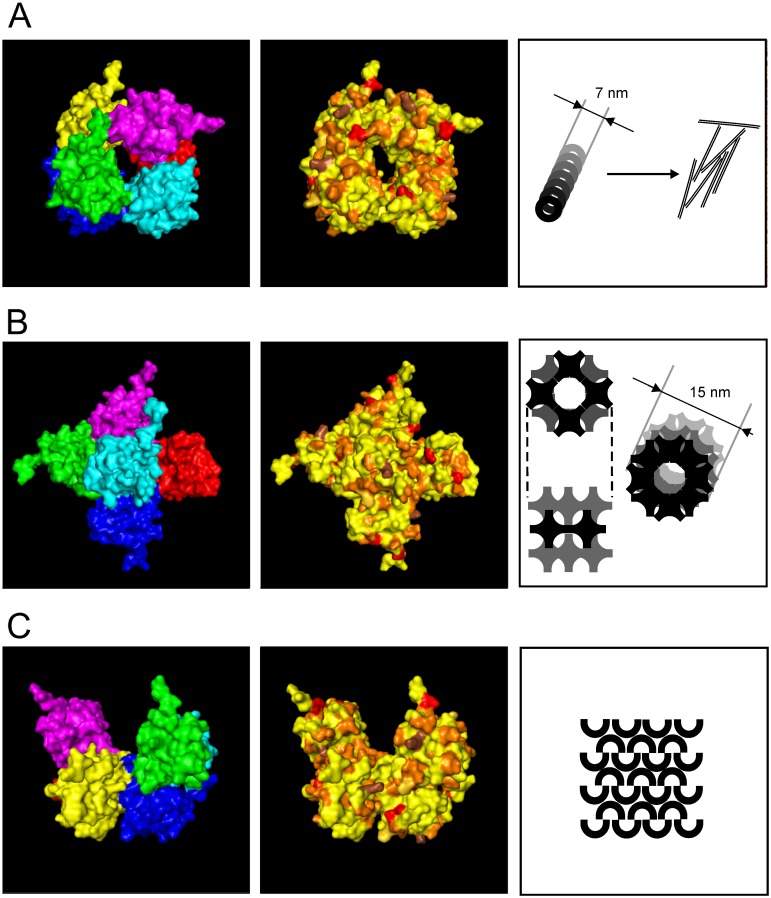
Predictions of possible assembly of six Hyd8 proteins showing the six proteins, amino acids coloured depending on properties and a located/postulated structure. Beside the six proteins in different colours, amino acids coloured depending on properties: yellow—hydrophobic, dark yellow—amphipathic, orange—polar, red—negatively charged, brown—positively charged. (A) A ring structure with 7 nm in diameter similar to HGFI from *Grifola frondosa* [17], (B) a plus-like structure which results in a 15 nm diameter if four plus structures are combined and (C) packing arrangement to a sheet-like layer such as HFBII from *Trichoderma reesei* [21].

Since hydrophobins on aerial hyphae are known to show a distinct rodlet structure, tertiary structure prediction was performed using the well studied spore wall hydrophobin DewA of *A*. *nidulans* as a template (Supporting Table 5 in S1 File). High similarity was observed for all hydrophobins, with short unaligned N- and C-termini (Supporting Figure 8 in S1 File). While there is little conservation of predicted α-helices, turns or ungrouped residues were seen and tertiary structure prediction showed the ability to interact for all hydrophobins, alone or in combination. Phenylalanine F86 of Hyd8 was active in forming a ring-like super-structure of 7 nm diameter composed of four hydrophobin molecules in one layer (Fig 5A, Supporting Figure 8 in S1 File), while F54 was able to initiate a plus-like structure of hexamers (Fig 5B, Supporting Figure 8 in S1 File). These plus-like subunits could assemble to a rodlet structure with a diameter of 15 nm. A sheet-like structure was additionally predicted from a packing arrangement (Fig 5C). The *in silico* analyses indicated the highest incidence for Hyd8 to form rodlet structures well supporting this hydrophobin as the major aerial hyphae coating molecule (Fig 4A). The remaining tertiary structures might instead be involved in hypha-hypha contact, host interaction or contact to other surfaces.

### Host recognition

*T*. *vaccinum* undergoes mutualistic symbiotic interactions with conifers, predominantly spruce and rarely pine. This interaction involves the close contact of plant and fungal surfaces in the soil environment. Hydrophobin expression patterns in response to root exudates, root volatiles and in mycorrhizal root tips of *P*. *abies* and *P*. *sylvestris* were obtained. Depending on the host plant, significant differences in expression profiles of hydrophobins were observed (Fig 4B–4E). For an early recognition of a potential host, root exudates induced *hyd4*, while *hyd9* was repressed with both hosts (Fig 4B). Compounds found on a regular basis within root exudates, IAA and ethanol, were tested and a significant induction was observed for *hyd8* and *hyd5* with ethanol at physiologically relevant concentrations of 0.01% and/or IAA (Fig 4C). Statistically significant induction was additionally detected for *hyd3* at 0.01 and 0.001 mM IAA. For *hyd4*, the highest induction was observed for ethanol, significantly decreasing with IAA addition.

Closer contact to the tree host was tested using root volatiles. This treatment led to an induction of *hyd5* with both hosts (Fig 4D). The gene *hyd4* was induced near *P*. *abies*, which is in contrast to the spruce root exudates tested above. After mycorrhiza was established, *hyd5* was induced in both interactions with significantly higher induction for the preferred host *P*. *abies* (Fig 4E). In addition, the aerial hyphae hydrophobin genes *hyd8* and *hyd9* were repressed.

### Fruiting body formation

*T*. *vaccinum* can produce fruiting bodies only in contact with a host tree. Therefore, we examined fruiting bodies collected near Jena. The homology of the fruiting body ITS sequence (KT315517) was 99% compared to *T*. *vaccinum* (FJ845444) and 92% for *T*. *vaccinum* GK6514 (AY573538) reflecting the known divergency within one species (see discussion). We examined all nine hydrophobin genes (without amplificates for *hyd6* and *hyd9*) and compared their expression to non-mycorrhizal mycelium of *T*. *vaccinum* GK6514 grown on compost soil to obtain patterns specific to fruiting body tissues and not responsive to the soil environment. Induction of *hyd3* and *hyd8* was detected (Fig 4F) with 93% and 87% sequence identity to *T*. *vaccinum* GK6514 Hyd3 and Hyd8 (Supporting Figure 4 in S1 File).

### Metal response in *T*. *vaccinum*

**Fig 6:**
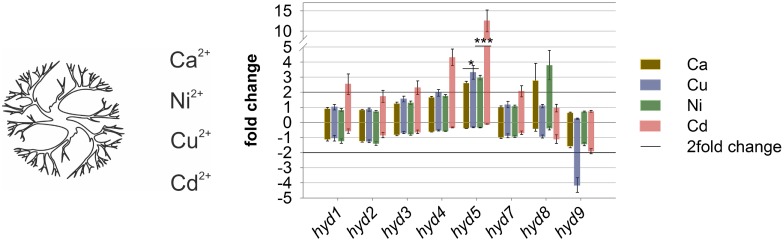
Relative expression levels of hydrophobin transcripts in mycelium treated with metals for 24 hours in liquid culture. Bars denote standard error. All values are fold changes compared to a control in liquid medium; significance level * *P* < 0.05, ** *P* < 0.005, *** *P* < 0.005.

Metal stress was tested, since cell wall adsorption could link metal tolerance to the cell surface and hence hydrophobins covering the hyphae. Exposure for 24 h was chosen to avoid morphological changes, e.g. production of aerial mycelium in long term cultivation. In all treatments, *hyd5* was induced, with significant differences between calcium and copper treatments and between nickel and cadmium treatments (Fig 6). For cadmium treated mycelium, a general induction of hydrophobin genes (*hyd1*, *hyd3*, *hyd4*, *hyd5* and *hyd7*) was seen. A specific reduction of *hyd9* was visible for the copper treatment.

## Discussion

Throughout the life cycle of *T*. *vaccinum*, the nine hydrophobin genes are differentially expressed (Fig 4). The total number corresponds to eight genes with *T*. *matsutake* or nine with *L*. *bicolor* 81306, while *L*. *bicolor* S238N-H82 carries twelve hydrophobin genes [28]. The latter fungus, however, has been found to also contain a high number of transposable elements accelerating evolution [28]. Differential expression of, among others, hydrophobin genes has been observed by Plett *et al*. [28], which led the authors to conclude that the hydrophobin gene family might have evolved with multiple members to aid the symbiotic lifestyle of ectomycorrhizal fungi. However, the number of hydrophobin genes in the first fungal species in which hydrophobins have been described, the white rot saprophytic basidiomycete *S*. *commune*, is even larger with 12 hydrophobin encoding genes [65]. The number of branches in our phylogenetic tree (Fig 2) indeed is high which might, just as indicated by Plett *et al*. [28], be a result of co-evolution in plant symbiosis.

The lowering of the surface tension by hydrophobins correlated with a role in aerial mycelium as shown for the *S*. *commune* aerial hyphae hydrophobin Sc3. Specifically for the up-regulated *hyd8*, hydrophobicity plots showed a similarity (Supporting Figure 3 in S1 File) that might indicate a similar function. The deletion of *sc3* in *S*. *commune* resulted in a flat phenotype [22], comparable to the substrate mycelium growing on compost soil with repressed *hyd8* supporting a functioning in aerial mycelium formation for this hydrophobin.

The regulation of *hyd4* and *hyd5* reflected host preference. In early steps of mycorrhization, after recognition of a potential host through root exudates, *hyd4* was induced (Fig 4B). This answer is refined in close contact through root volatiles, where only the preferred host induced *hyd4* and significantly improved *hyd5* expression (Fig 4D). The hydrophobin expression profiles of *hyd4* and *hyd5* with respect to host specificity could explain, as one factor in the complex gene regulation, the differences in duration for host colonization with different host trees, i.e. few weeks for development of compatible mycorrhiza and three to four months for low compatibility interactions (Fig 4E). With respect to host specificity, the regulation of *hyd4* through exudates may be linked to plant defence.

The net charge profile of Hyd4 (Supporting Figure 6 in S1 File), indicative of surface charge, was similar to that observed for *P*. *tinctorius* HydPt-1 also regulated early in mycorrhization [66]. The changes in the pH versus net charge profile may be linked to a function in the adaptation of the hyphal surface to the prevailing conditions in the rhizosphere, where steep gradients from acidic root surfaces to soil occur [67, 68]. The regulatory motif for Rim101, known from *Saccharomyces cerevisiae* for adaptation to alkaline conditions, and from *A*. *nidulans* PacC involved in pH response, might be associated with transcription factor binding in *T*. *vaccinum* as well. Additionally, the hydrophobicity pattern and phylogenetic analysis of Hyd5 showed similarity to LbH9 of *L*. *bicolor* [28] and Hyd1 of *T*. *terreum* (Fig 2 [69]).

The final step in the life cycle of a mycorrhizal fungus is the production of fruiting bodies as place of sexual spore formation. Hydrophobins like *S*. *commune* Sc4 are specifically expressed in fruiting bodies to avoid water clogging of gas channels [70]. Similarity to *T*. *vaccinum* proteins is seen with hydrophobicity patterns of Hyd3 and Hyd8 (Supporting Figure 3 in S1 File), both induced in fruiting bodies (Fig 4F). The role of Hyd8 in general decoration of aerial hyphae would be in line with decorating the aerial hyphae of the pileus. Since only fruiting bodies, not all aerial mycelium, contain gas channels, the differential expression pattern of Hyd3 would coincide with its lining gas channels within fruiting bodies.

The ability of hydrophobins to form rodlet structures was connected with decoration of hyphae. Using *in silico* analysis, the potential formation of three distinct structures by all hydrophobins of *T*. *vaccinum* was demonstrated (Fig 5). Interestingly, one region of the hydrophobins was highly variable and could be decisive to create hydrophobins with different properties in evolution (Supporting Figure 8 in S1 File). The proteins with the longest variable bend were the aerial mycelium induced hydrophobins (genes *hyd3*, *hyd4*, *hyd8*) and Hyd9 (Supporting Figure 8 in S1 File). The main aerial hydrophobin with the longest bend, Hyd8, was shown to potentially form rodlets of expected sizes from ring-like structures [24]. Rodlets of the plus-like structures are more complex, with four of these subunits assembling to one layer and then forming rodlets by stacking (Fig 5B). For the *N*. *crassa* protein Eas, stacking by the Cys3-Cys4 loop was shown to allow rodlet formation [23]. However, the *T*. *vaccinum* Cys3-Cys4 loop seems too short for the interaction and stacking like in Eas. The third prediction shows similarity to the crystal structure of HFBII, which forms a sheet-like layer (Fig 5C [21]). The result is a two dimensional network of proteins which could occur, for example, at an interface where its viscoelastic properties could add to the stability of adhesion. As with many different hydrophobin arrangements observed [20], the *T*. *vaccinum* hydrophobins represent a modular system to establish contact between surfaces.

Mycorrhizal fungi are able to enhance metal resistance of their host [71]. During metal exposure, regulation of hydrophobin gene expression had been shown before [31, 72, 73]. Potentially, protection of the hyphae by increasing hydrophobicity and hence lowering uptake of toxic metals through Hyd5 could play a role (Fig 6).

The ancestral host with ectomycorrhiza has been debated for some time. For *Tricholoma*, we could show that an angiosperm plant is the likely ancestral host as *Fagus* and *Quercus* were revealed by two independent methods to be the likely ancestral host trees (Supporting Figure 7 in S1 File). Prior studies postulated independent origins for ECM with angiosperms and gymnosperms [74]. Host changes and loss of symbiosis have occurred repeatedly within the Agaricales [75]. Surface interactions with the host through hydrophobins, located at the interface between plant and fungus, are likely involved in host switching. Adaptation to a host might be the cause for nucleotide changes in the Hyd5 group after gene amplification. The *hyd4* sequences showed even higher numbers of exchanges between different members of this subgroup (see Fig 2), which implies that the first root contact needs less adaption as it seems based on a less conserved hydrophobin function. Both Hyd4 and Hyd5, in contrast to general hydrophobins Hyd1 or Hyd8, showed accelerated evolution (d_n_/d_s_ > 1) and a relaxed or positive selection, *e*.*g*. for host adaption. As seen earlier [7], ITS sequence divergence between *T*. *vaccinum* isolates was found; in this study *T*. *vaccinum* below *P*. *abies* near Jena (Germany) and *T*. *vaccinum* FJ845444 from Canada is 1%, while 8% were found between *T*. *vaccinum* GK6514 and a strain (AY573538) from Nassreith, Austria.

**Fig 7:**
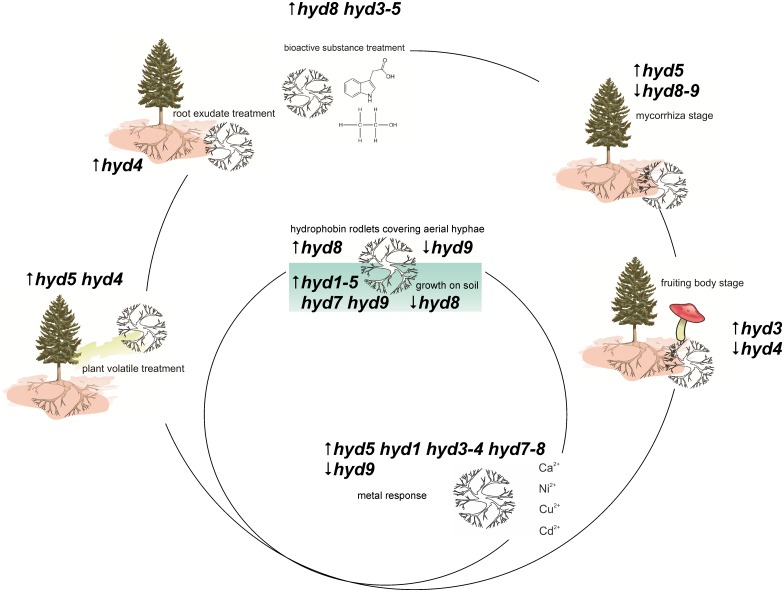
Summary of differentially expressed hydrophobins in life cycle of *T*. *vaccinum*.

Our extensive expression profiling thus could provide insight into the evolution and function of hydrophobins. It suggests an important role of the different hydrophobins for aerial mycelium, fruiting body and ectomycorrhiza establishment and functioning (Fig 7).

## Supporting Information

S1 File **S1 Table. *Tricholoma* species used. S2 Table. NCBI accession numbers of hydrophobin gene sequences. S3 Table. Oligonucleotides used. S4 Table. Host preference in *Tricholoma* species. S5 Table. C-score values of the 3D structure prediction via I-TASSER. S1 Fig. Experimental setup of fungus-root-interaction.** (A) Root exudates collection system using a sterile pipette tip box: a seedling (*P*. *abies* or *P*. *sylvestris*), b 20% MMNa solution (B) Split Petri dish system for volatile experiment: a seedling (*P*. *abies* or *P*. *sylvestris*), b fungus (*T*. *vaccinum*), c MMNa, d half concentrated MMNb, e cellophane membrane, f 2nd Petri dish to inclose possible volatiles. (C) Axenic Petri dish system to synthesize ectomycorrhiza: a seedling (*P*. *abies* or *P*. *sylvestris*), b fungus (*T*. *vaccinum*), c MMNa, d open area, e cellophane membrane. **S2 Fig. Regulation of the reference genes *act1* and *tef1*.** Fold changes of relative expression after cultivation for either 16 days (a) or 32 days (b) on Pachlewski [39], Moser B [40], BAF [41] and ½ concentrated MMNb media. **S3 Fig. Hydrophobicity plot of hydrophobins from *T*. *vaccinum*.** (A) Hydrophobins are compared to class I (B) with Sc3 of *Schizophyllum commune* (P16933), Eas of *Neurospora crassa* (AAB24462) and DewA of *Aspergillus nidulans* (P52750) and class II (C) with Hfb2 of *Trichoderma reesei* (P79073), ZtH1 of *Zymoseptoria tritici* (XP_003849840) and NC2 of *N*. *crassa* (4AOG_A). **S4 Fig. Hydrophobin alignment.** Secretion signal peptides and signal sequence proteases are indicated by a triangle, identical amino acids shaded in grey, conservative exchanges outlined in black. **S5 Fig. Consensus phylogram of basidiomycete hydrophobins.** Gene and protein sequences (protein IDs according to JGI annotations and NCBI accession numbers) are used from *Tricholoma vaccinum* (red), *Tricholoma matsutake*, *Tricholoma terreum*, *Coprinopsis cinerea*, *Heterobasidion annosum*, *Schizophyllum commune*, *Pisolithus tinctorius*, *Laccaria bicolor* and *Paxillus involutus*. Bayesian posterior probability values are shown above corresponding branches; branch lengths are proportional to evolutionary distances. The class II PbHyd2 from the ascomycete *Paracoccidioides brasiliensis* was included as outgroup. **S6 Fig. The pH versus net charge (Z) plots of hydrophobins.** *T*. *vaccinum* (TvHyd: AHZ18297, AHZ18298, AHZ18299, AHZ18300, AHZ18301, AHZ18303, AHZ18304, AHZ18305), *T*. *matsutake* (TmHyd: 1252927, 1291850, 1386086, 1383591, 1422529, 254042, 1403957, 1383622), *P*. *tinctorius* (Hyd-Pt1 P52748), *S*. *commune* (Sc3 P16933), *L*. *bicolor* (LbHyd9 XP_001885701), and *H*. *annosum* (Hah2 ABA46363). **S7 Fig. A consensus phylogram of *Tricholoma* species based on ITS sequences from NCBI and UNITE.** Marked are the three groups which represent fruiting body cap colour in brown, yellow, and grey. In addition the host-mycobiont status is given in the matrix on the right: compatible in green, low compatibility in light yellow, incompatible in white, unknown status in grey. Bayesian posterior probability values are shown above corresponding branches. Branch lengths are proportional to evolutionary distances. The species *T*. *stiparophyllum* was set as outgroup. **S8 Fig. Multiple 3D alignment of hydrophobins calculated by Matras.** (A) Multiple alignment of protein sequences with the average amino acid sequence (Avg. Seq., small letters represent consensus with threshold of 50–99%, capital letters represent 100% consensus), their location on the surface (O) or inside (I) the protein and average secondary structure (Sec. Str.: H, α-helix; T, hydrogen bonded turn; S, bend and C, any residue that does not belong to any of the previous groups) and (B) superimposed structure of all nine hydrophobins of *T*. *vaccinum*; left lateral view, right top view. Coloured regions in A and B show similarity in tertiary structure. Position of variable bend, F54 (active in plus-like structure) and F86 (active in ring-like structure) are labelled bold for Hyd8.
(PDF)
