# Supplementary material for: Hydrophobins in the Life Cycle of the Ectomycorrhizal Basidiomycete Tricholoma vaccinum
Source: PLoS One. 2016 Dec 9;11(12):e0167773. doi: 10.1371/journal.pone.0167773 (PMC5147985; doi:10.1371/journal.pone.0167773)
Supplement: S1 File — S1 Table. Tricholoma species used. S2 Table. NCBI accession numbers of hydrophobin gene sequences. S3 Table. Oligonucleotides used. S4 Table. Host preference in Tricholoma species. S5 Table. C-score values of the 3D structure prediction via I-TASSER. S1 Fig. Experimental setup of fungus-root-interaction. (A) Root exudates collection system using a sterile pipette tip box: a seedling (P. abies or P. sylvestris), b 20% MMNa solution (B) Split Petri dish system for volatile experiment: a seedling (P. abies or P. sylvestris), b fungus (T. vaccinum), c MMNa, d half concentrated MMNb, e cellophane membrane, f 2nd Petri dish to inclose possible volatiles. (C) Axenic Petri dish system to synthesize ectomycorrhiza: a seedling (P. abies or P. sylvestris), b fungus (T. vaccinum), c MMNa, d open area, e cellophane membrane. S2 Fig. Regulation of the reference genes act1 and tef1. Fold changes of relative expression after cultivation for either 16 days (a) or 32 days (b) on Pachlewski [39], Moser B [40], BAF [41] and ½ concentrated MMNb media. S3 Fig. Hydrophobicity plot of hydrophobins from T. vaccinum. (A) Hydrophobins are compared to class I (B) with Sc3 of Schizophyllum commune (P16933), Eas of Neurospora crassa (AAB24462) and DewA of Aspergillus nidulans (P52750) and class II (C) with Hfb2 of Trichoderma reesei (P79073), ZtH1 of Zymoseptoria tritici (XP_003849840) and NC2 of N. crassa (4AOG_A). S4 Fig. Hydrophobin alignment. Secretion signal peptides and signal sequence proteases are indicated by a triangle, identical amino acids shaded in grey, conservative exchanges outlined in black. S5 Fig. Consensus phylogram of basidiomycete hydrophobins. Gene and protein sequences (protein IDs according to JGI annotations and NCBI accession numbers) are used from Tricholoma vaccinum (red), Tricholoma matsutake, Tricholoma terreum, Coprinopsis cinerea, Heterobasidion annosum, Schizophyllum commune, Pisolithus tinctorius, Laccaria bicolor and Paxillus involutus. Bayesian posterior prob [file pone.0167773.s001.pdf]

Supporting Information

S1 Table. *Tricholoma* species used.

Strain	Number	Resource
T. albobruneum	MG091013-05	Microbial Communication (FSU Jena)
T. fracticum	DG11114	Microbial Communication (FSU Jena)
T. fulvum	FSU4732	Jena Microbial Resource Collection
T. fulvum	FSU10089	Jena Microbial Resource Collection
T. imbricatum	FSU4738	Jena Microbial Resource Collection
T. terreum	FSU4735	Jena Microbial Resource Collection
T. ustaloides	FSU4739	Jena Microbial Resource Collection
T. vaccinum	Fruiting body	+50° 55’ 11.29, +11° 31’ 30.15
T. vaccinum	MG111121-03	Microbial Communication (FSU Jena)
T. vaccinum	GK6514 (FSU4731)	Microbial Communication (FSU Jena)

S2 Table. NCBI accession numbers of hydrophobin gene sequences.

hyd1	*hyd2*	*hyd3*	*hyd4*	*hyd5*	*hyd6*	*hyd7*	*hyd8*	*hyd9*
KM486624	KM486625	KM486626	KM486627	KM486628	-	KM486629	KM486630	-
-	KM486631	KM486632	KM486633	-	-	-	-	-
KM486634	KM486635	-	-	-	-	KM486636	KM486637	-
KM486638	a:KM486639<br>b:KM486640	-	KM486641	-	-	KM486642	KM486643	-
KM486644	KM486645	KM486646	-	-	-	-	-	-
AY048578[26]	KM486652	-	-	-	-	-	KM486653	-
KM486654	KM486655	-	KM486656	-	-	KM486657	KM486658	-
KM486659	KM486661	KM486660	KM486664	KM486662	KM486663	KM486665	KM486659	-
a:KM486647<br>b:KM486648	KM486649	-	KM486650	-	-	KM486651	-	-
KJ507742	KJ507743	KJ507744	KJ507745	KJ507746	KJ507747	KJ507748	KJ507749	KJ507750
1253242	1292165	1386401	1383906	1422844	254042	1404272	1383937	-

S3 Table. Oligonucleotides used.

Transcript	Fwd-and Rev-primer (5’- 3’)	Efficiency (%)
tef1	GGCAACTTATTGTTGCTGTGAACAA	95
	GACCTTCTTGATAAAGTTGGAGGTT	
act1	ACAACCATGTTCCCCGGTATCT	91
	TTCGCTCAGGAGGAGCAACAAT	
hyd1	ATTGGTCTTGGGAGTGGCTC	98
	ACAAGTTGACAGGAGAGCACC	
hyd2	AACCTCCAGGGTATTACCGG	106
	TCTCGCAGCAGACAGGTTGT	
hyd3	TGACTTCGGTCGGGTTTGAC	95
	CTTGGTTGCAGCAGACAGGTT	
hyd4	GGTTACCACCGTTGGTTTCAAC	95
	CAGCAGACTGGTTGTGAAGCA	
hyd5	AATCTCTTACTGCCTCTATTGGTAC	99
	GCAGCAGACGGGTTGTTGA	
hyd7	CTCCTGTTGCCAGTGCGTTTT	102
	AGGATAGAAGTATCTTGGTGGTTC	
hyd8	TCTCCAGAATGTAGTCACCACC	105
	GCAGACTGGTTGGGTGGC	
hyd9	GTTCTCAAAAGTCGCTCTTTTCG	108

	TTACAAAATTTGCTCGTTGATAGGG	
hyd1-fb	CAGTGCACTCCTATCTCTGTC	-
	GTAATGGTTGTTGGTGCAGCAG	
hyd2-fb	GTTACTGGTGACGTTGGTACC	-
	TTGCTGCAGCAAACGGGTTG	
hyd3-fb	TTTGGTGACTTCAGTTGGTTTTGA	-
	ATAGGCTTGGTTGCAGCAGAC	
hyd4-fb	CTTGTTGGCGCCCCCATC	-
	GCTGCCTGCAGCACCAC	
hyd5-fb	TAGTTGGCCTCAACGTCCAAG	-
	AGCAGACGGGTTGTTGAGAG	
hyd7-fb	CTTCGATCTTGGTTGGCATGC	-
	GTAGGTATTGTTTTCACAGCAAACAG	
hyd8-fb	GTCGCTGGCGCCACGA	-
	CACCGCTGCCTGCAGC	
hyd1ampl	CGCTGCTTCTCCTGTCCCAG	-
	CAAGTTGACAGGAGAGCACCCG	
hyd2ampl	CTTTGTCGCCGCCACTCCCA	-
	GTTCACAGGAGAGCAGCCAACC	
hyd3ampl	CATGCCTGGTGTGCAACTTACG	-
	GCTAATGAGACCGCCTGAAAAGC	
hyd4ampl	CAATGGAGGACCCATCCCTGG	-
	GGGATGCAGCCGTTGCCG	
hyd5ampl	CCACCCCCATCCCCGAC	-
	CTACAAATTGATGGGAGAGCAACCAA	
hyd7ampl	CCTCACCGTCGTGCAACTCC	-
	GGTTGTTGCGAGCTAGAAGACG	
hyd8ampl	CTCGTTTCTGGAGGACCCATTC	-
	ACAAGGCCACCTGAAGACGAAC	
hyd9ampl	GTTCTCAAAAGTCGCTCTTTTCG	-
	TTACAAAATTTGCTCGTTGATAGGG	

S4 Table. Host preference in *Tricholoma* species.

Species	*Picea*	*Pinus*	*Fagus*	*Betula*	*Salix*	*Larix*	*Popolus*	*Quercus*
T. acerbum	A*	A	B	A	A	A	A	E
T. aestuans	F	A	A	A	A	A	A	A
T. albobrunneum	B	E	A	A	A	A	A	A
T. alboconicum	-	-	-	-	-	-	-	-
T. album	A	A	B	E	A	A	A	B
T. apium	F	A	A	A	A	A	A	A
T. argyraceum	B	B	C	C	B	A	A	B
T. atrosquamosum	C	C	C	A	A	A	A	A
T. aurantium	D	C	A	A	A	A	A	A
T. batschii	B	E	A	A	A	A	A	A
T. bufonium	C	A	D	A	A	A	A	B
T. caligatum	D	D	A	A	A	A	A	A
T. cingulatum	A	A	A	A	F	A	A	A
T. columbetta	C	A	D	B	A	A	A	B
T. dulciolens	-	-	-	-	-	-	-	-
T. equestre	B	E	A	A	A	A	A	A
T. flavovirens	B	E	A	A	A	A	A	A

T. focale	A	F	A	A	A	A	A	A
T. fracticum	B	E	A	A	A	A	A	A
T. fucatum	D	D	A	A	A	A	A	A
T. fulvocastaneum	F	A	A	A	A	A	A	A
T. fulvum	A	A	A	E	A	A	A	B
T. gausapatum	A	F	A	A	A	A	A	A
T. imbricatum	A	F	A	A	A	A	A	A
T. inocybeoides	B	B	C	C	B	A	A	B
T. lascivum	A	A	E	A	A	A	A	B
T. myomyces	B	D	B	B	B	A	A	A
T. nictitans	F	A	A	A	A	A	A	A
T. orirubens	B	B	D	A	A	A	A	B
T. pardinum	B	A	D	A	A	A	A	B
T. pessundatum	B	E	A	A	A	A	A	A
T. populinum	A	A	A	A	A	A	F	A
T. portentosum	B	E	A	A	A	A	A	A
T. psammopus	A	A	A	A	A	F	A	A
T. pseudonictitans	F	A	A	A	A	A	A	A
T. saponaceum	A	A	E	A	A	A	A	B
T. scalpturatum	B	B	C	B	A	A	A	B
T. sciodes	A	A	F	A	A	A	A	A
T. sejunctum	C	B	C	A	A	A	A	B
T. squarrulosum	C	C	C	A	A	A	A	A
T. stans	B	E	A	A	A	A	A	A
T. stiparophyllum	A	A	B	E	A	A	A	A
T. sulfurescens	A	A	D	A	A	A	A	C
T. sulfureum	B	B	D	A	A	A	A	B
T. terreum	B	E	A	A	A	A	A	A
T. triste	B	D	B	B	B	A	A	A
T. umbonatum	A	A	F	A	A	A	A	A
T. ustale	A	A	E	A	A	A	A	B
T. ustaloides	A	A	C	A	A	A	A	D
T. vaccinum	E	B	A	A	A	A	A	A
T. virgatum	D	B	B	A	A	A	A	A
T. viridilutescens	A	A	F	A	A	A	A	A

* A, 0 %; B, 1-24 %; C, 25-49 %; D, 50-74 %; E, 75-99 %; F, 100 %

S5 Table. C-score values of the 3D structure prediction via I-TASSER.

Hydrophobin	default	EAS	HFB2	DewA
1	-1.14	-0.21	-0.78	-0.022
2	-0.95	-0.20	-0.56	-0.17
3	-1.41	-0.73	-1.10	-0.33
4	-0.90	-1.10	-0.99	-0.35
5	-1.16	-1.00	-0.76	-0.21
7	-0.99	-0.55	-0.49	+0.01
8	+0.08	-0.70	-0.55	+0.53
9	-0.07	-0.75	-0.65	+0.32

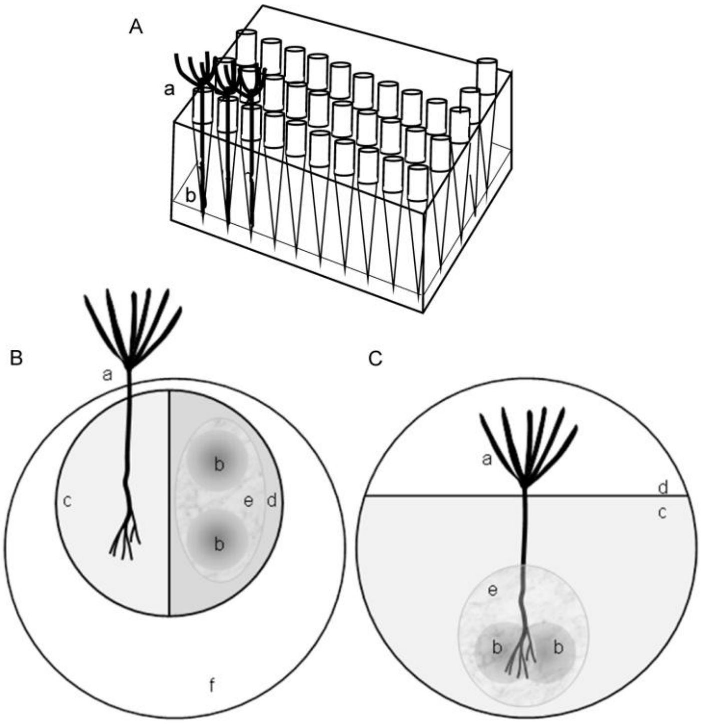

S1 Fig. Experimental setup of fungus-root-interaction. (A) Root exudates collection system using a sterile pipette tip box: a seedling (*P. abies* or *P. sylvestris*), b 20 % MMNa solution (B) Split Petri dish system for volatile experiment: a seedling (*P. abies* or *P. sylvestris*), b fungus (*T. vaccinum*), c MMNa, d half concentrated MMNb, e cellophane membrane, f 2nd Petri dish to inclose possible volatiles. (C) Axenic Petri dish system to synthesize ectomycorrhiza: a seedling (*P. abies* or *P. sylvestris*), b fungus (*T. vaccinum*), c MMNa, d open area, e cellophane membrane.

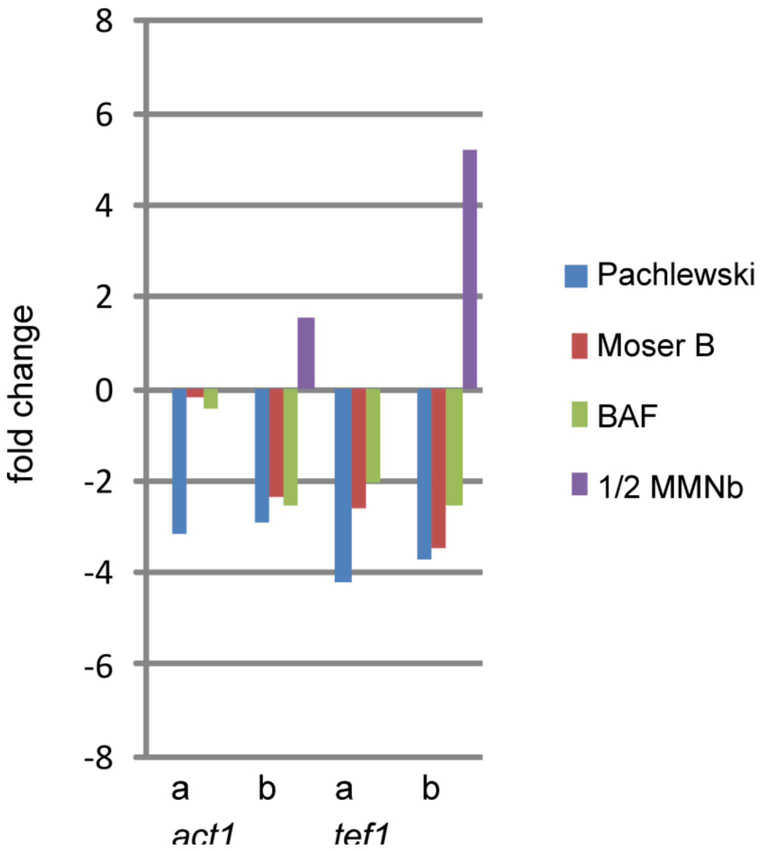

S2 Fig. Regulation of the reference genes *act1* and *tef1*. Fold changes of relative expression after cultivation for either 16 days (a) or 32 days (b) on Pachlewski [39], Moser B [40], BAF [41] and ½ concentrated MMNb media.

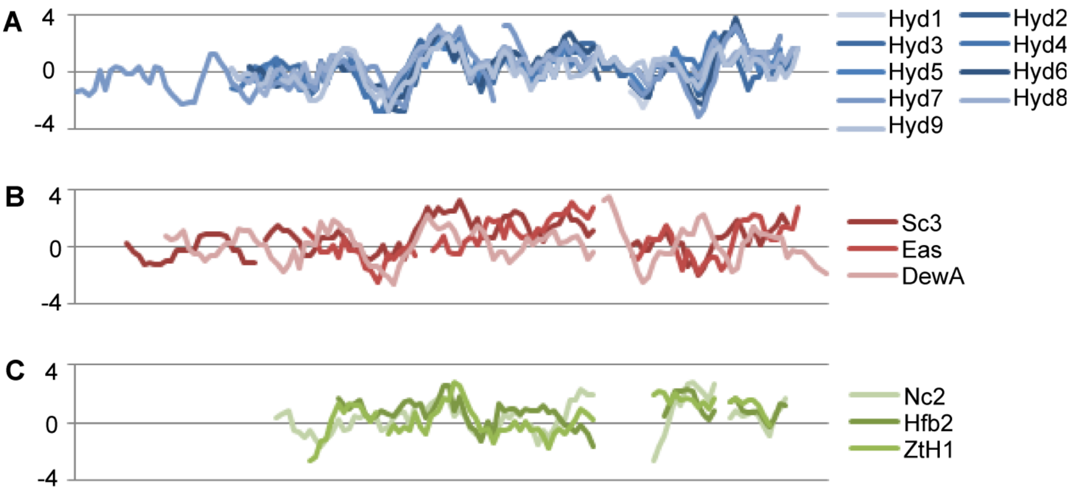

S3 Fig. Hydrophobicity plot of hydrophobins from *T. vaccinum*. (A) Hydrophobins are compared to class I (B) with Sc3 of *Schizophyllum commune* (P16933), Eas of *Neurospora crassa* (AAB24462) and DewA of *Aspergillus nidulans* (P52750) and class II (C) with Hfb2 of *Trichoderma reesei* (P79073), ZtH1 of *Zymoseptoria tritici* (XP_003849840) and NC2 of *N. crassa* (4AOG_A).

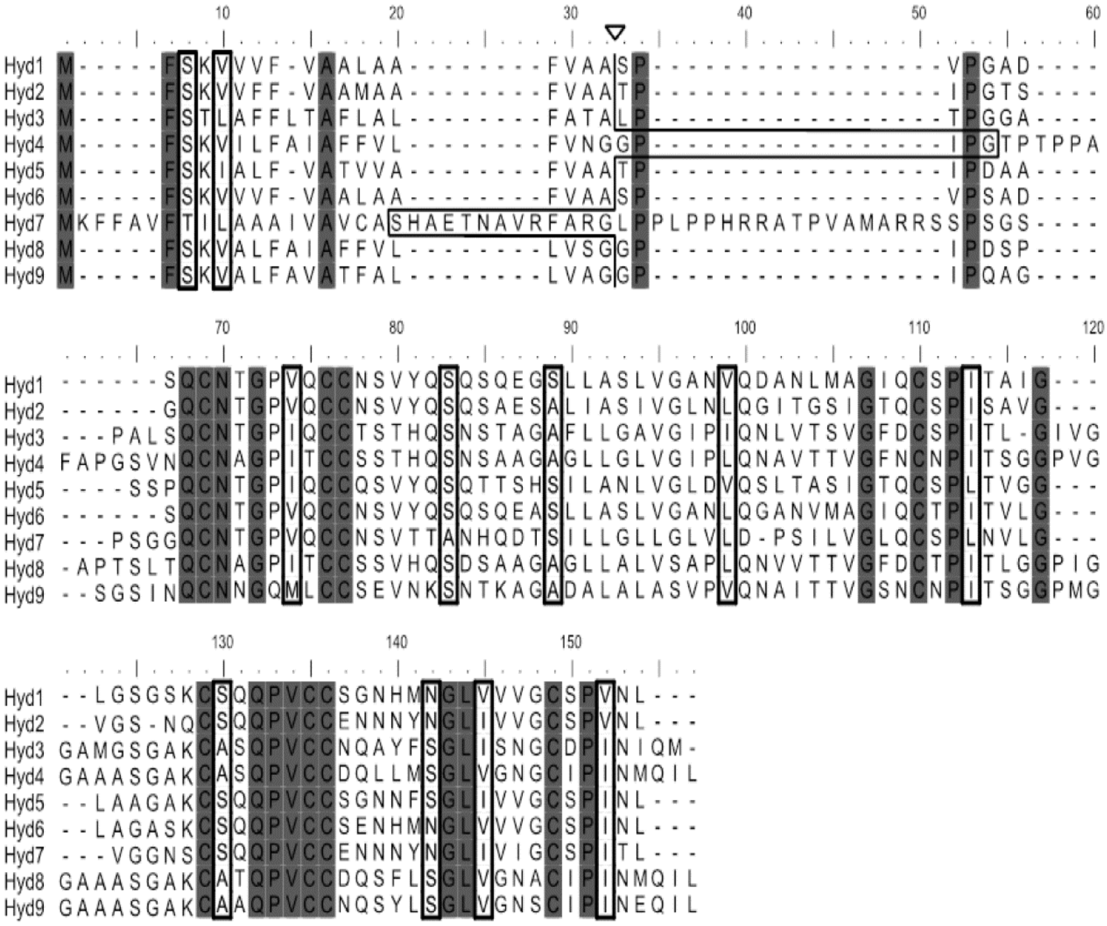

S4 Fig. Hydrophobin alignment. Secretion signal peptides and signal sequence proteases are indicated by a triangle, identical amino acids shaded in grey, conservative exchanges outlined in black.

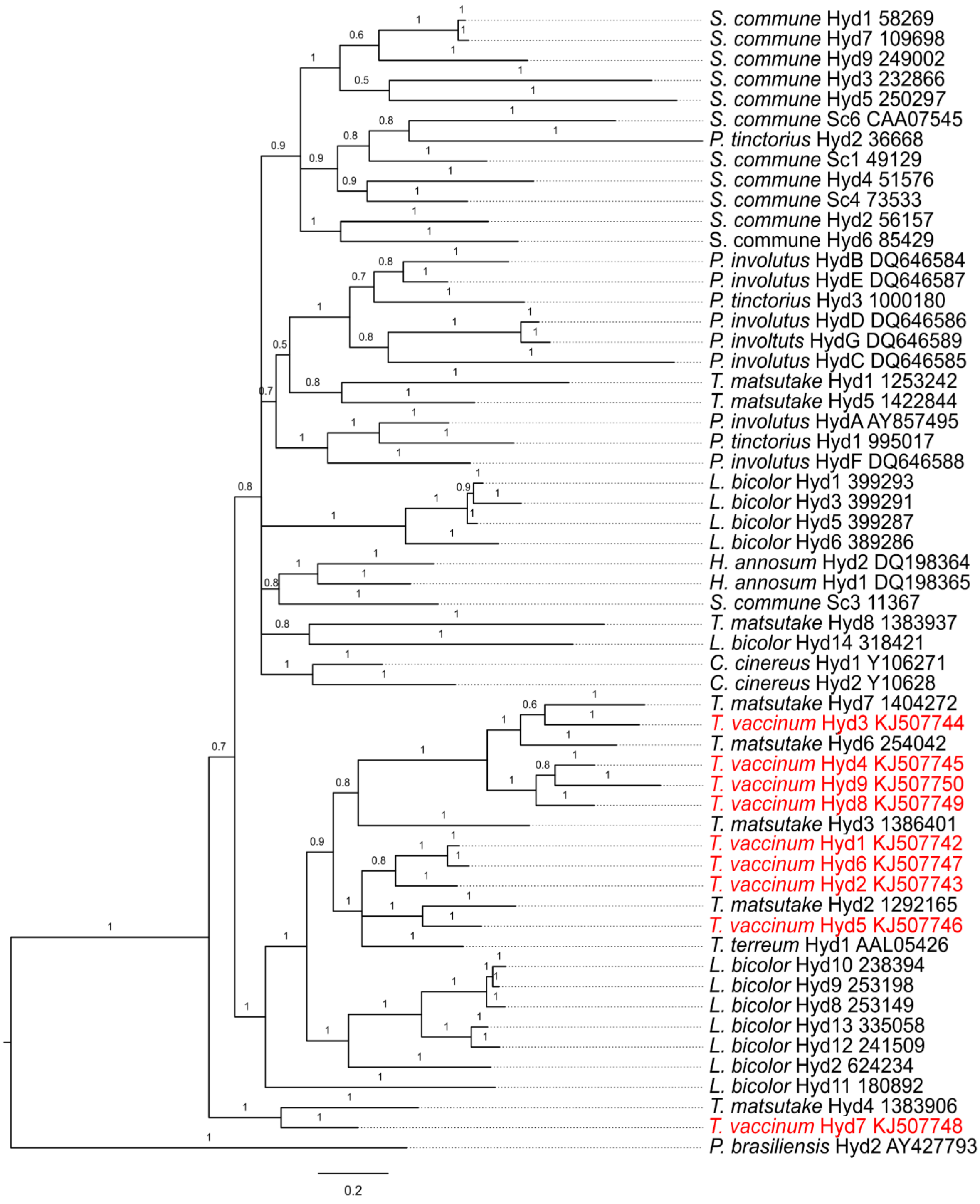

S5 Fig. Consensus phylogram of basidiomycete hydrophobins. Gene and protein sequences (protein IDs according to JGI annotations and NCBI accession numbers) are used from *Tricholoma vaccinum* (red), *Tricholoma matsutake, Tricholoma terreum, Coprinopsis cinerea, Heterobasidion annosum, Schizophyllum commune, Pisolithus tinctorius, Laccaria bicolor* and *Paxillus involutus*. Bayesian posterior probability values are shown above corresponding branches; branch lengths are proportional to evolutionary distances. The class II PbHyd2 from the ascomycete *Paracoccidioides brasiliensis* was included as outgroup.

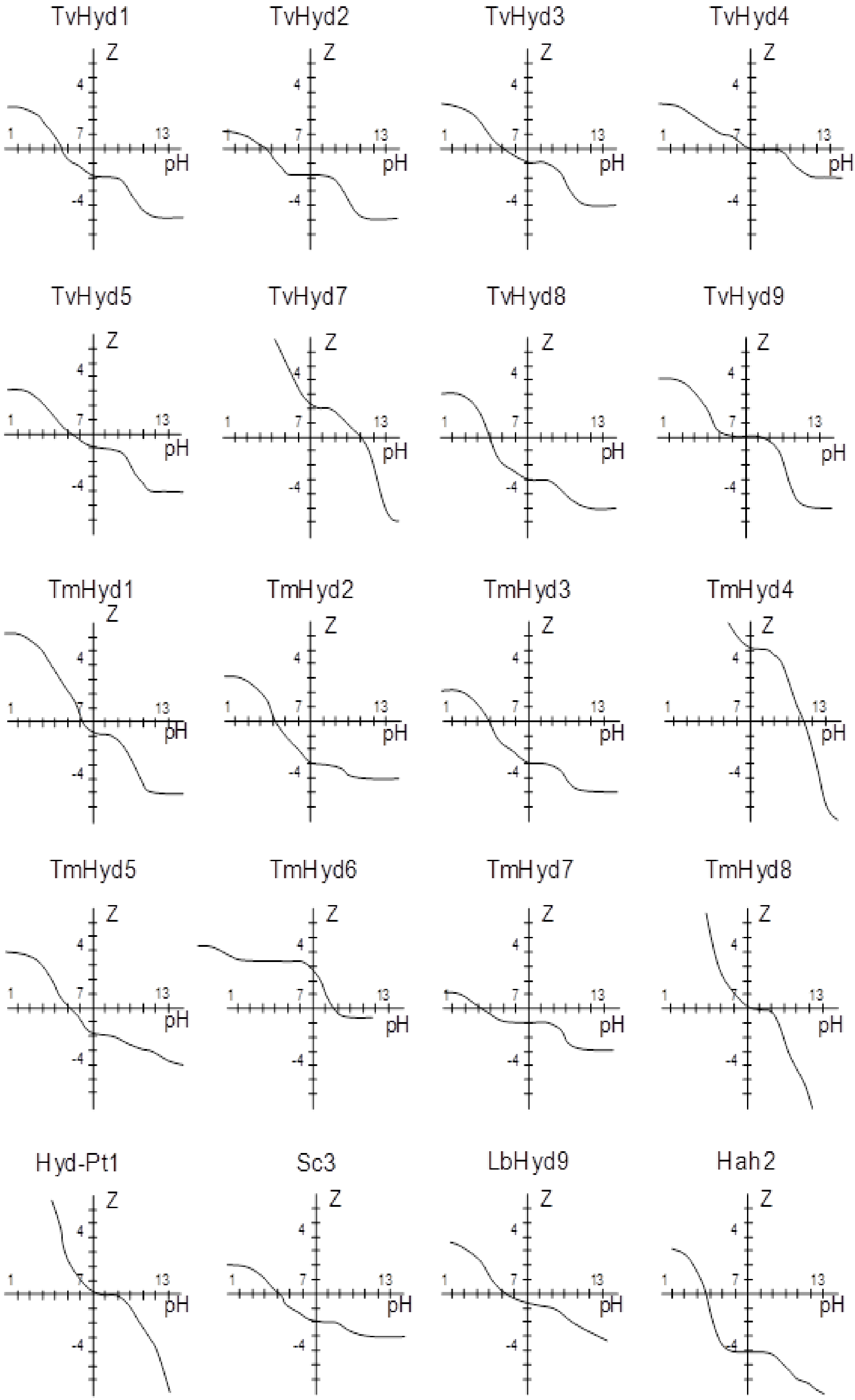

S6 Fig. The pH versus net charge (Z) plots of hydrophobins. *T. vaccinum* (TvHyd: AHZ18297, AHZ18298, AHZ18299, AHZ18300, AHZ18301, AHZ18303, AHZ18304, AHZ18305), *T. matsutake* (TmHyd: 1252927, 1291850, 1386086, 1383591, 1422529, 254042, 1403957, 1383622), *P. tinctorius* (Hyd-Pt1 P52748), *S. commune* (Sc3 P16933), *L. bicolor* (LbHyd9 XP_001885701), and *H. annosum* (Hah2 ABA46363).

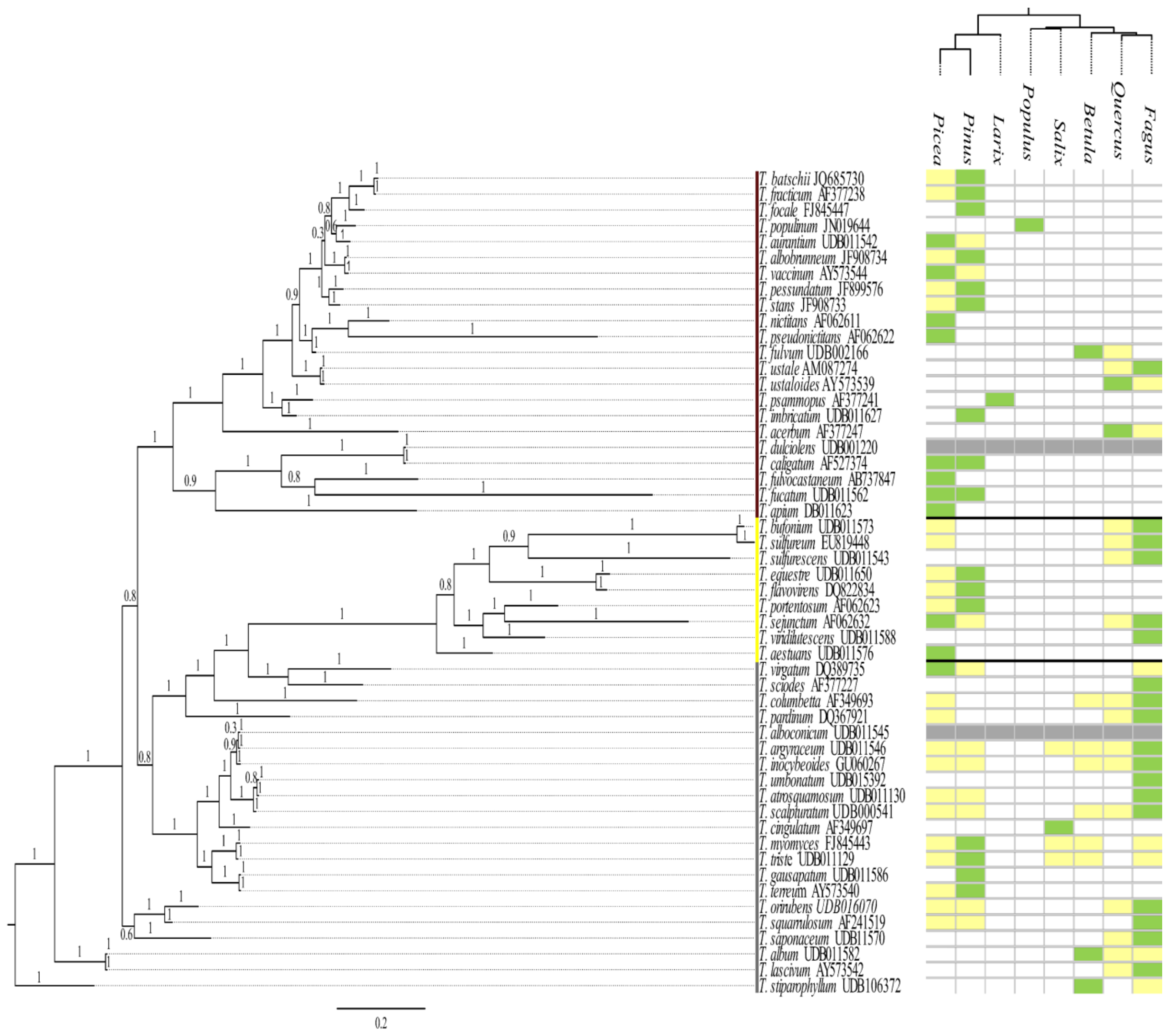

S7 Fig. A consensus phylogram of *Tricholoma* species based on ITS sequences from NCBI and UNITE. Marked are the three groups which represent fruiting body cap colour in brown, yellow, and grey. In addition the host-mycobiont status is given in the matrix on the right: compatible in green, low compatibility in light yellow, incompatible in white, unknown status in grey. Bayesian posterior probability values are shown above corresponding branches. Branch lengths are proportional to evolutionary distances. The species *T. stiparophyllum* was set as outgroup.

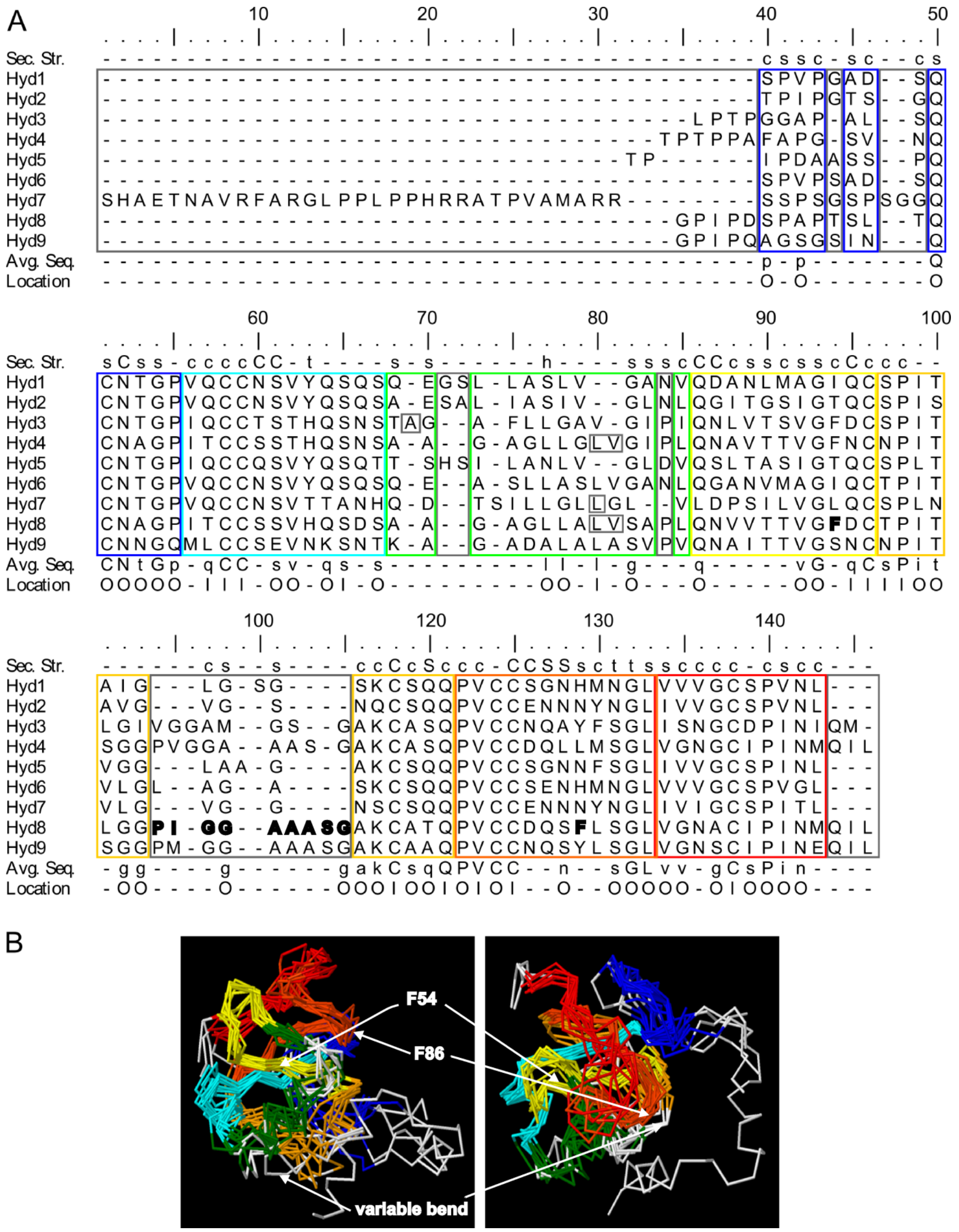

S8 Fig. Multiple 3D alignment of hydrophobins calculated by Matras. (A) Multiple alignment of protein sequences with the average amino acid sequence (Avg. Seq., small letters represent consensus with threshold of 50-99 %, capital letters represent 100 % consensus), their location on the surface (O) or inside (I) the protein and average secondary structure (Sec. Str.: H, α-helix; T, hydrogen bonded turn; S, bend and C, any residue that does not belong to any of the previous groups) and (B) superimposed structure of all nine hydrophobins of *T. vaccinum*; left lateral view, right top view. Coloured regions in A and B show similarity in tertiary structure. Position of variable bend, F54 (active in plus-like structure) and F86 (active in ring-like structure) are labelled bold for Hyd8.
